# Enhancer promoter interactome and Mendelian randomization identify network of druggable vascular genes in coronary artery disease

**DOI:** 10.1186/s40246-022-00381-4

**Published:** 2022-03-04

**Authors:** Arnaud Chignon, Samuel Mathieu, Anne Rufiange, Déborah Argaud, Pierre Voisine, Yohan Bossé, Benoit J. Arsenault, Sébastien Thériault, Patrick Mathieu

**Affiliations:** 1 Laboratory of Cardiovascular Pathobiology, Department of Surgery, Institut de Cardiologie Et de Pneumologie de Québec, Quebec Heart and Lung Institute/Research Center, Laval University, 2725 Chemin Ste-Foy, Québec, QC G1V-4G5 Canada; 2 Department of Surgery, Laval University, Quebec, Canada; 3 Department of Molecular Medicine, Laval University, Quebec, Canada; 4 Department of Medicine, Laval University, Quebec, Canada; 5 Department of Molecular Biology, Medical Biochemistry and Pathology, Laval University, Quebec, Canada

**Keywords:** Atherosclerosis, Coronary artery disease, Genes, 3D genome, Mendelian randomization, Druggable genome, Network

## Abstract

Coronary artery disease (CAD) is a multifactorial disorder, which is partly heritable. Herein, we implemented a mapping of CAD-associated candidate genes by using genome-wide enhancer-promoter conformation (H3K27ac-HiChIP) and expression quantitative trait loci (eQTL). Enhancer-promoter anchor loops from human coronary artery smooth muscle cells (HCASMC) explained 22% of the heritability for CAD. 3D enhancer-promoter genome mapping of CAD-genes in HCASMC was enriched in vascular eQTL genes. By using colocalization and Mendelian randomization analyses, we identified 58 causal candidate vascular genes including some druggable targets (*MAP3K11*, *CAMK1D*, *PDGFD*, *IPO9* and *CETP*). A network analysis of causal candidate genes was enriched in TGF beta and MAPK pathways. The pharmacologic inhibition of causal candidate gene *MAP3K11* in vascular SMC reduced the expression of athero-relevant genes and lowered cell migration, a cardinal process in CAD. Genes connected to enhancers are enriched in vascular eQTL and druggable genes causally associated with CAD.

**Supplementary Information:**

The online version contains supplementary material available at 10.1186/s40246-022-00381-4.

## Introduction

Coronary artery disease (CAD) is a complex trait disorder and a leading cause of morbidity-mortality. The dysregulated expression of genes and activation of pathways culminate in the development of vascular atheromatous plaque, a hallmark feature of CAD [1]. Functional assessment and cell tracking experiments have shown that vascular smooth muscle cells are recruited to the plaque where they play a significant role in the development of CAD [2]. The identification of molecules involved in the pathophysiology of CAD could lead to the development of novel therapies. However, the discovery of disease-associated drug targets is limited by several factors, which include limited information about the disease process and indirect evidence obtained from animal and cell experiments [3]. Also, epidemiological studies carried out in humans by measuring biomarkers or intermediate molecules is subject to bias and reverse causality [4]. As such, only a small fraction of drug development programs leads to licensed drugs [5].

Genetic association studies (GWAS) have underlined that CAD is heritable (narrow sense heritability estimated at 40–50%) and involves several loci (~ 160 identified so far) [6]. Genetic association data provides a rich resource, which may help identify key targets involved in the development of disorders. Gene variants associated with intermediate phenotypes (e.g. gene expression) and the disease can be leveraged to assess causality similarly to a randomized clinical trial [7]. According to Mendel’s second law of random allocation of alleles, independent instrumental variables (IVs) (uncorrelated variants) can be assessed to mimic the effect of drugs (exposure, e.g. as determined by gene expression) on the risk of disorders (outcome). The use of multiple genetic variants that are associated with the exposure can be used as IVs in Mendelian randomization (MR) to assess causal associations for the risk of disorders [8]. Since the allocation of alleles is random and occurs before the development of the outcome, MR technique is not prone to bias and reverse causation [9]. Studies have underlined that pharmacological targets supported by genetics have a much higher chance of success during the different phases of drug development [10].

Despite rigorous preclinical screening, the failure of some compounds is related to drug-related adverse side-effects, which are often discovered in large phase 3 randomized clinical trials (RCTs) [11]. This may result in substantial monetary loss with the attrition of resources for the development of novel drug pipelines [12]. Target-related pleiotropy is one cause of such failure [10]. For instance, a drug target with opposite directional effect on two major outcomes may negate potential benefit. Interrogation of large electronic health record databases in genotyped individuals provides a resource to assess hundreds of traits and disorders. The assessment of risk variants linked to a target in a phenome-wide analysis study (PheWAS) is thus a strategy to evaluate potential side-effects of drug-gene pairs [13]. Also, the assessment of putative causal candidate drug targets for their association with monogenic disorders by using large resources where data are collated, such as in the Human Phenotype Ontology, is another approach to assess drug-related safety issues [14, 15].

Complex systems in which gene expression and interaction of molecules are establishing a trajectory to health or disease are increasingly investigated by the integration of data in network [16]. The network topology is highly modular and provides information for distinctive molecules, which interact together to drive different functions [17]. As such, genes highly connected tend to be enriched in essential functions and in drug targets [18, 19]. The implementation of network to assess molecules with impact on the biological pathways relevant to a disease is a useful strategy to prioritize genes and to address the function of the whole system and its components (modules).

In order to identify causal genes by using genome-wide association studies (GWAS), we need to map variants to potential gene targets. Mapping of genes is compounded by several factors, which includes the linkage disequilibrium (LD) between the variants (the extent to which variants are correlated) and the fact that the vast majority of gene variants associated with complex traits and disorders reside in the noncoding genome [20]. Variants within the noncoding genome are enriched in active regulatory regions such as distant acting enhancers and may impact on the expression in *cis* (locally) through the conformation of chromatin [21]. Genome-wide assessment of chromatin conformation based on Hi-C and its derivatives (e.g. HiChIP) has revealed that chromatin looping between enhancers and promoters provides regulatory mechanisms to control gene expression [22]. Growing evidence suggests that a hierarchy among the distant regulatory regions provides another layer of control on gene expression [23]. To this effect, highly connected hub enhancers in 3D communities regulate lineage-specific genes [24]. In addition, the architecture of chromatin conformation is largely cell specific [25]. Hence, GWAS mapping using genome-wide 3D data provides an additional layer of information to identify putative causal genes in disease-relevant cells [26]. Another strategy to map the genetic variants to potential targets is to assess the associations with expression quantitative trait loci (eQTL) derived from disease-relevant tissue(s) [27]. Herein, we implemented an integrative approach including enhancer-promoter chromatin conformation and eQTL mapping, causal inference, interrogation of the druggable genome and network biology to capture GWAS-associated molecules and pathways, which could be targeted pharmacologically. Specifically, we assessed whether enhancer-promoter chromatin looping in vascular smooth muscle cells explained a significant proportion of the heritability for CAD and if it was enriched in vascular eQTL genes. Genome-wide mapping using enhancer-promoter conformation and eQTL data were integrated in a framework to assess causal associations and to prioritize druggable genes with the help of networks.

## Results

### Tissue enrichment and pathway analyses

**Fig. 1:**
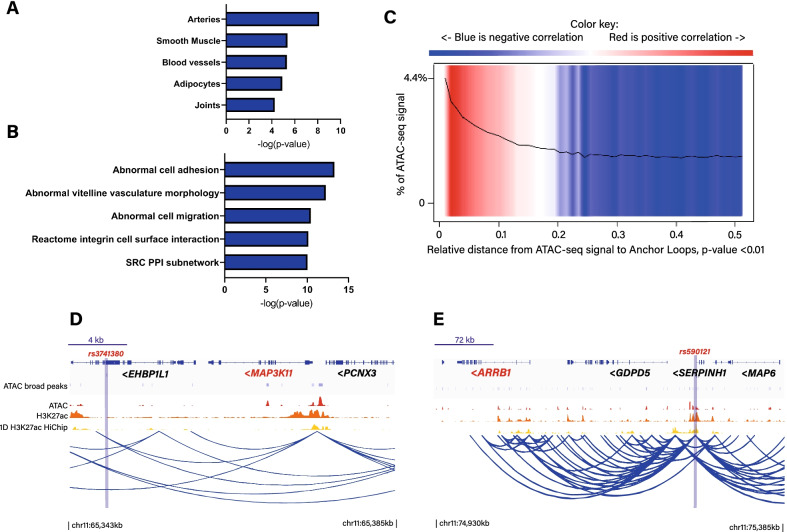
Enrichment and enhancer-promoter mapping. **A**, **B** Tissue and pathway enrichment of CAD GWAS. **C** Heatmap representing the relative distance of ATAC-seq signal to enhancer-promoter anchor loops; correlation is the measure of the observed to expected relative distance to the query point. **D**, **E** Individual independent significant SNPs and 3D mapping in enhancer-promoter HiChIP; tracks represent genes, ATAC-seq, H3K27ac ChIP, 1D H3K27ac-HiChIP and arcs of significant loops; vertical bar represents the SNP of interest and mapped genes are in red

Summary-level data of meta-analysis from UK Biobank (UKB) and CARDIoGRAMplusC4D including 123,733 CAD cases and 424,528 controls [28] was leveraged to assess tissue and pathway enrichment. We implemented Data-driven Expression Prioritized Integration for Complex Traits (DEPICT) to document the enrichment of tissue and pathways for genetic association data in CAD. By using GWAS summary statistics, DEPICT provides an analysis in which risk loci mapped genes are integrated to a vast collection of human tissue gene expression to prioritize highly expressed genes and their relevant pathways. According to DEPICT, genetic association for CAD was enriched in arteries (*P* = 7.38 × 10^–9^), smooth muscle (*P* = 4.33 × 10^–6^) and blood vessels (*P* = 4.77 × 10^–6^) (Fig. 1A) (Additional file 1: Table S1). We next evaluated the enrichment of genetic association in pathways. DEPICT showed an enrichment for abnormal cell adhesion (*P* = 5.04 × 10^–14^), abnormal vitelline vasculature morphology (*P* = 5.64 × 10^–13^), abnormal cell migration (*P* = 3.67 × 10^–11^), integrin cell surface interactions (*P* = 7.37 × 10–^11^) and src PPI subnetwork (*P* = 9.31 × 10^–11^) (Fig. 1B) (Additional file 2: Table S2). These data thus indicated that the vasculature and smooth muscle cells as well as their related functions such as adhesion and migration are key features associated with CAD-gene variants.

### Heritability for CAD explained by the vascular enhancer-promoter connectome

Considering the high enrichment of genetic association data for CAD in smooth muscle and arteries, we analyzed publicly available HiChIP for H3K27ac obtained in human coronary artery smooth muscle cells (HCASMC) (GSE101498). H3K27ac-HiChIP provides a high-definition map of chromatin conformation between enhancers and promoters [29]. After a stringent loop call with FitHiChIP (FDR < 1 × 10^–6^), we identified 224,209 confident loops in HCASMC. The anchor loops were significantly enriched in open chromatin detected by assay for transposase-accessible chromatin and sequencing (ATAC-seq) in HCASMC (fold enrichment = 2.9, *P* < 2.2 × 10^–16^, binomial test) (Fig. 1C). By using HOMER, a pathway enrichment of anchor loops in HCASMC using the Kyoto Encyclopedia of Genes and Genomes (KEGG) showed an enrichment for endocytosis (*P* = 1.36 × 10^–10^), regulation of actin cytoskeleton (*P* = 1.70 × 10^–8^), phosphatidylinositol signaling system (*P* = 5.43 × 10^–8^) and autophagy (*P* = 5.54 × 10^–8^) (Additional file 3: Table S3). We next wondered whether enhancer-promoter contacts in HCASMC explained a significant part of the heritability in CAD risk. For that purpose, we partitioned the heritability of *cis*-anchor loops in HCASMC by using the full baseline model of 53 annotations in the stratified LD score regression framework (Methods) [30]. This analysis revealed that chromosomal interactions in HCASMC were enriched for regions that explained the heritability of CAD (enrichment = 2.38, *P* = 5.90 × 10^–5^) (Additional file 4: Table S4). Despite representing only 9% of CAD-single nucleotide polymorphisms (SNPs), variants within anchor loops in HCASMC explained 22% of the heritability for CAD (Additional file 4: Table S4).

### Identification of gene promoters connected to CAD associated variants within distant enhancers

To identify gene promoters mapped by chromatin looping in H3K27ac-HiChIP in HCASMC, we identified lead and individual significant SNPs (P_GWAS_ < 5 × 10^–8^, *r*^2^ < 0.6) that overlapped with enhancer loops (Methods). In total, 353 individual significant SNP-enhancer-promoter loop pairs tagging 228 gene promoters were identified (Additional file 5: Table S5). The mean number of loops per gene promoter was 1.5 and the average distance between enhancers and gene promoters was ~ 168 kb. For instance, at 11q13.1, rs3741380, a CAD individual significant SNP intronic to *EHBP1L1*, is localized within an interacting loop with the promoter of *MAP3K11* located ~ 32 kb downstream (Fig. 1D). We next hypothesized that CAD gene variants may be enriched in hub enhancers having a high level of chromatin contacts (in network with a degree ≥ 90th percentile) (Methods). In HCASMC, we identified 5,455 highly connected hub enhancers involved in chromatin looping. This analysis showed a significant enrichment of CAD individual significant SNPs associated with hub enhancers (observed/expected = 3.4, *P* = 1.9 × 10^–6^, binomial test). Among the different genes mapped by CAD variants and enhancer-promoter looping, *SH2B3* is a gene connected to a hub enhancer. *SH2B3* has been previously identified as a causal candidate gene for CAD [31]. On the other hand, some genes identified by chromatin looping were not previously mapped in CAD. For instance, at 11q13.4, rs590121 is located in a distant hub enhancer intronic to *SERPINH1* and having contacts with multiple enhancers-promoters including with the promoter of *ARRB1* located ~ 298 kb upstream (Fig. 1E).

### Identification of vascular eQTLs

We next examined if CAD-associated genes mapped by enhancer-promoter chromatin looping were also significant vascular expression quantitative trait loci (eQTL). CAD-individual significant SNPs were mapped to eQTLs of the aorta in GTEx v8. In total, 15,516 CAD SNP-eQTL gene pairs were significant at FDR 5% in the aorta and tagged 202 genes (Additional file 6: Table S6). Among the 228 genes mapped by enhancer-promoter chromatin looping there were 41 genes (18%), which were also CAD-associated eQTL genes (fold enrichment observed/expected = 21.4, *P* < 2.2 × 10^–16^, binomial test). Hence, these findings highlight that gene mapping of CAD genetic association data with enhancer-promoter chromatin conformation in HCASMC is enriched in vascular eQTL genes.

### Genetic colocalization analyses

**Fig. 2:**
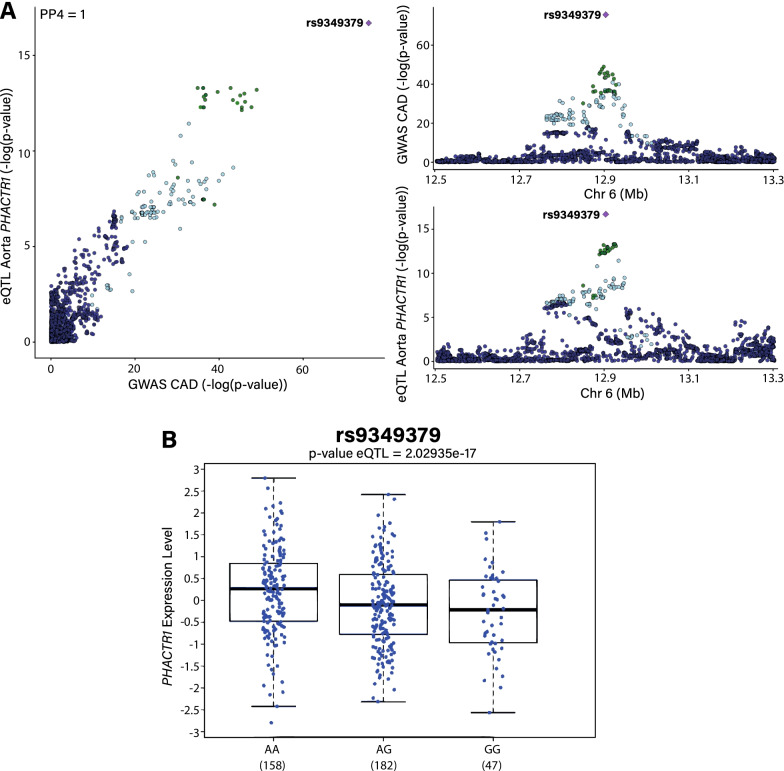
Colocalization and vascular eQTL. **A** LocusCompare plot showing –log *P*-values for CAD-GWAS and eQTL in the aorta (GTEx v8) at the *PHACTR1* locus. **B** eQTL in the aorta (GTEx v8) for rs9349379

By combining enhancer-promoter chromatin looping and eQTL data, there were 383 genes mapped by CAD-associated SNPs. We performed Bayesian colocalization analyses between the eQTL signal (GTEx v8) of the 383 mapped genes in the aorta with the GWAS signal for CAD. We identified 35 genes with a with a high posterior probability (PP) (PP > 0.8) of shared signal between eQTLs in the aorta and CAD-GWAS (*CDH13, PHACTR1, TCF21, N4BP2L2, SYPL2, TWIST1, PDGFD, IPO9, FHL3, UTP11, GGCX, SEMA5A, DMPK, MIA3, TMEM133, CAMK1D, ARHGAP42, DAGLB, DMWD, CETP, MORF4L1, JCAD, MFGE8, HAPLN3, HHIPL1, DHX36, B3GNT8, BMP1, LMOD1, FAM117B, MAT2A, ATP2B1, EXOSC5, EIF2B2, ZEB2*) (Additional file 7: Table S7). Genes with a shared signal were enriched in gene ontology (GO) for nervous system development (*P* = 1.24 × 10^–4^) negative regulation of cell adhesion (*P* = 1.99 × 10^–4^) and positive regulation of endothelial cell proliferation (*P* = 2.18 × 10^–4^) (Additional file 8: Table S8). Figure 2A shows a LocusCompare plot for the GWAS associations at 6p24.1 where there is a shared signal (PP = 1) with the expression of *PHACTR1*, a gene previously identified as a causal candidate at this locus [32]. Rs9349379 is the strongest SNP for both GWAS and eQTL in the aorta for the expression of *PHACTR1*, a gene encoding for a regulator of actin polymerization [33]. Allele G-rs9349379 (EUR freq = 0.40) increases the risk of CAD (OR: 1.11, 95% CI 1.10–1.12, *P*_GWAS_ = 2.71 × 10^–76^) and is associated with a decreased expression of *PHACTR1* in the aorta (Fig. 2B). This analysis underlined some targets identified in previous screen and functional assays such as *CDH13, TCF21, LMOD1* and *JCAD* [34–37], but also identified novel potential causal candidates such as *EXOSC5* and *B3GNT8*, which encode for a RNA exosome component and a galactosyltransferase respectively.

### Mendelian randomization

**Fig. 3:**
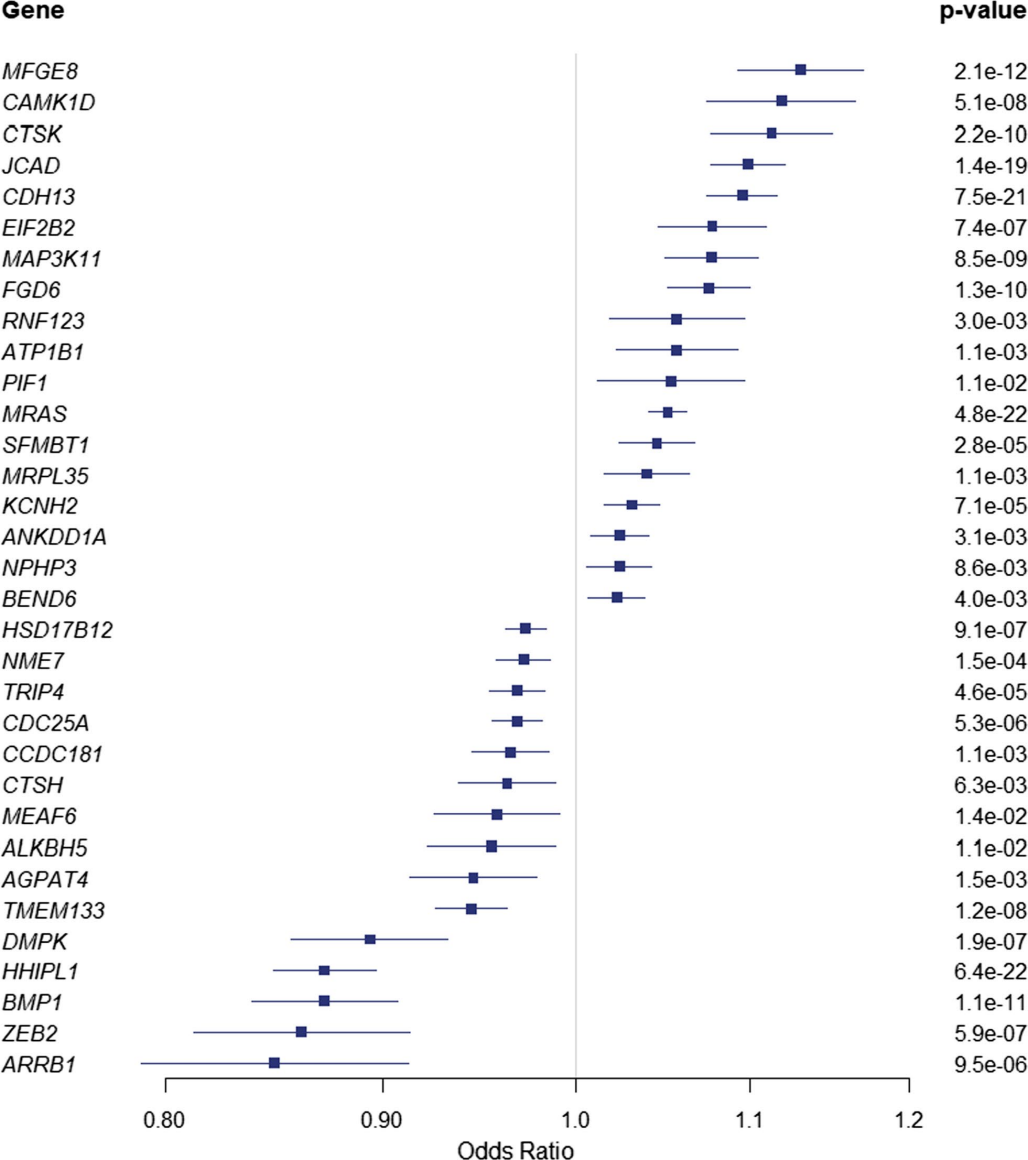
Mendelian randomization. Odds ratio and 95% CI for vascular genes (aorta) and CAD risk. Data are for 1SD

In order to further evaluate causal associations, we implemented a 2-sample Mendelian randomization (MR) between gene expression in the aorta (GTExV8) by using a minimum of three independent (*r*^2^ < 0.1) *cis*-variants as IVs and genetic associations for CAD as the outcome. For that purpose 3D and eQTL mapped genes were evaluated in MR. In inverse variance weighted (IVW) MR, we identified 74 vascular genes expressed in the aorta that were significantly associated with CAD (FDR < 0.05). Among these genes, 33 did not show heterogeneity (Cochran’s *Q* test > 0.05) and were considered as causally associated with CAD (*MRAS, HHIPL1, CDH13, JCAD, MFGE8, BMP1, FGD6, CTSK, MAP3K11, TMEM133, CAMK1D, DMPK, ZEB2, EIF2B2, HSD17B12, CDC25A, ARRB1, SFMBT1, TRIP4, KCNH2, NME7, ATP1B1, MRPL35, CCDC181, AGPAT4, RNF123, ANKDD1A, BEND6, CTSH, NPHP3, PIF1, ALKBH5, MEAF6*) (Fig. 3) (Additional file 9: Table S9). These 33 vascular genes were enriched in GO for cardiac muscle cell membrane repolarization (*P* = 1.44 × 10^–4^), proteolysis involved in cellular catabolic process (*P* = 4.80 × 10^–4^) and positive regulation of potassium ion transmembrane transport (*P* = 2.74 × 10–4) (Additional file 10: Table S10). Among the causally associated vascular genes, there were 10 genes also identified in colocalization analyses (PP > 0.8) (*HHIPL1, CDH13, JCAD, MFGE8, BMP1, TMEM133, CAMK1D, DMPK, ZEB2, EIF2B2*). In total, by using colocalization and MR approaches, we identified 58 potential causal vascular genes for CAD (Additional file 11: Table S11). Among the causal candidate genes, 9 were identified by both eQTL and enhancer-promoter conformation mapping (*FHL3, MIA3, MAT2A, GGCX, DAGLB, MAP3K11, ATP2B1, EXOSC5, B3GNT8*), whereas 6 genes (*MEAF6, AGPAT4, KCNH2, ARRB1, PIF1, UTP11*) were mapped only by chromatin conformation. For instance, *AGPAT4*, a gene encoding for a lysophosphatidic acid acyltransferase, is negatively associated with the CAD risk in MR (OR: 0.94, 95% CI 0.91–0.97, *P*_causal_ = 1.5 × 10^–3^). At this locus, two CAD independent significant SNPs (rs9295142, rs142697177) located in an intronic enhancer of *AGPAT4* are within a chromatin loop with the promoter of the same gene (Additional file 19: Figure S1).

In sensitivity analysis, we assessed causal associations by using weighted median MR, which provides a robust assessment with up to 50% of invalid instrumental variables (variants with horizontal pleiotropy) [38]. Among the 33 vascular genes associated with CAD in IVW MR, we found that 29 genes (*MFGE8, CAMK1D, CTSK, JCAD, CDH13, EIF2B2, MAP3K11, FGD6, RNF123, ATP1B1, SFMBT1, MRPL35, MRAS, KCNH2, NPHP3, ANKDD1A, BEND6, CTSH, NME7, CCDC181, HSD17B12, TRIP4, CDC25A, TMEM133, DMPK, ZEB2, ARRB1, BMP1, HHIPL1*) were also significant in weighted median MR analysis (Additional file 12: Table S12). The directional effects were concordant between IVW and weighted median MR analyses.

### Network and prioritization

**Fig. 4:**
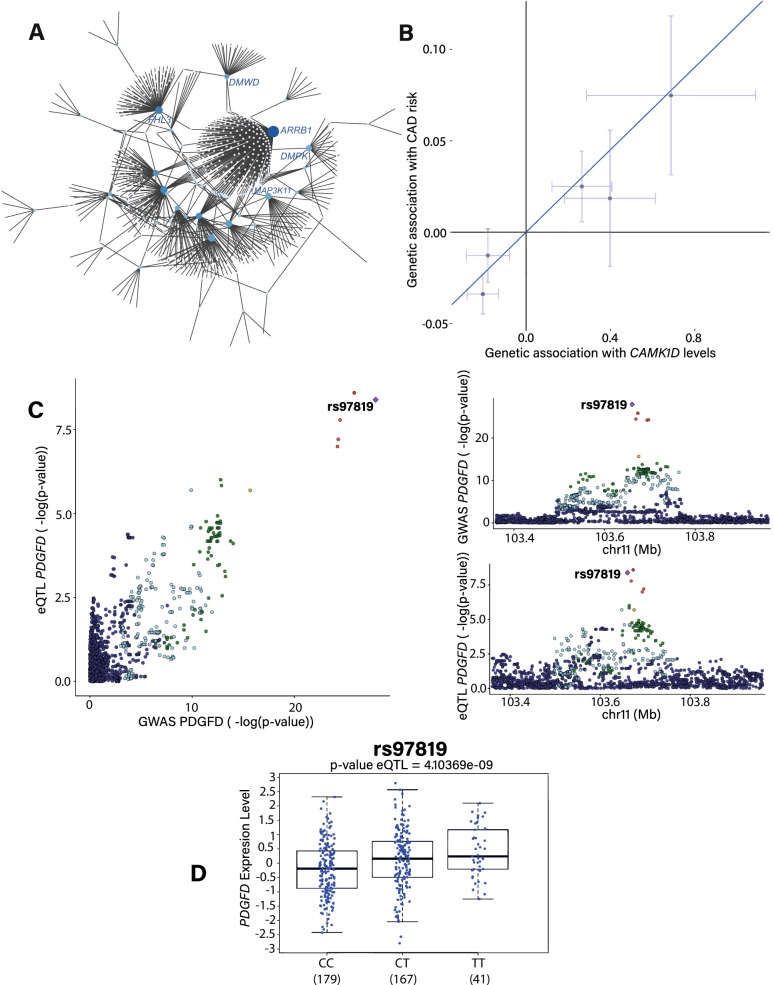
Network and causally associated vascular genes. **A** Protein interactions derived network extracted from causally associated vascular genes. **B** IVW MR for *CAMK1D*. **C** LocusCompare plot showing –log *P*-values for CAD-GWAS and eQTL in the aorta (GTEx v8) at the *PDGFD* locus. **D** eQTL in the aorta (GTEx v8) for rs97819

We aimed to characterize putative causal vascular genes singled out by the colocalization and MR screens by using a network approach with the objectives to identify: (1) key driver genes and (2) pathways of connected genes with functional relevance. A network was constructed from the DifferentialNet dataset, which includes 134,223 protein interactions, and inferred to the aorta from the gene expression profile (Methods) [39]. Candidate causally associated vascular genes identified by MR and colocalization analyses were used as seeds to generate the network, which encompassed 681 nodes (genes) and 763 edges (connections) (Fig. 4A). A pathway enrichment analysis showed that genes within the network were enriched in TGF-beta signaling pathway (*P* = 1.87 × 10^–20^), signaling events mediated by HDAC class I (*P* = 3.46 × 10^–17^), and MAPK signaling pathway (*P* = 1.62 × 10^–16^) (Additional file 13: Table S13). Causally associated genes were enriched in nodes with a high degree (degree > 90th percentile) (fold enrichment 3.83, *P* = 2.70 × 10^–25^, hypergeometric test). Genes with a high degree and acting as hub in networks are enriched in drug targets and are involved in key biological functions. Predicted CAD causally associated vascular genes in MR and acting as hub gene (high degree in PPI network) include among others *ARRB1* (OR: 0.84, 95%CI: 0.78–0.91, *P*_causal_ = 9.46 × 10^–6^), *CDC25A* (OR: 0.96, 95% CI 0.95–0.98, *P*_causal_ = 5.33 × 10^–6^), *KCNH2* (OR: 1.03, 95% CI 1.01–1.04, *P*_causal_ = 7.14 × 10^–5^) and *MAP3K11* (OR: 1.07, 95%CI: 1.05–1.10, *P*_causal_ = 8.46 × 10^–9^).

### Druggability of candidate causal genes

We next assessed whether causally associated vascular genes in CAD were potentially druggable by using The Drug Gene Interaction Database (DGIdb) [40]. DGIdb includes an exhaustive list of drug-gene pairs, which are collated from different resources. In total 383 compounds targeting 13 predicted causally associated vascular genes were identified by DGIdb (*CTSK, MAP3K11, CDC25A, ARRB1, KCNH2, ATP1B1, PDGFD, IPO9, GGCX, DAGLB, CETP, DHX36, CAMK1D*) (Additional file 14: Table S14). Considering the directional effects in MR, 5 vascular genes are potential targets for drug inhibition (*CTSK, MAP3K11, KCNH2, ATP1B1, CAMK1D).* For instance, the vascular expression of *CAMK1D* which encodes for a calcium calmodulin dependent protein kinase, is positively associated with the risk of CAD in MR (OR: 1.11, 95% CI 1.07–1.16, *P*_causal_ = 5.12 × 10^–8^) (Fig. 4B). Several drugs under investigation are kinase inhibitors, which are reported to target CAMK1D. By using colocalization analyses, the directional effects of the candidate causal SNP between vascular gene expression and CAD-risk suggest that the inhibition of 4 genes may lower disease-associated risk (*PDGFD, IPO9, GGCX, CETP).* As an example, among the potential drug targets, *PDGFD* encodes for platelet derived growth factor D. In the aorta, colocalization between eQTL for *PDGFD* and CAD-GWAS (PP = 0.99) suggests a causal association and prioritizes gene variant rs974819 (Fig. 4C). In the aorta, T-rs974819 (EUR freq = 0.72) is associated with a higher expression of *PDGFD* (Fig. 4D) and an elevated CAD-risk (OR: 1.06, 95% CI 1.05–1.07, *P*_GWAS_ = 1.11 × 10^–28^). According to the directional effects in MR and colocalization data, 4 genes (*CDC25A, ARRB1, DAGLB*, *DHX36*) could be targeted by agonist-based therapy. However, for these targets there is no agonist compound reported in DGIdb.

As some potential disease-associated targets may be linked with adverse side-effects, we undertook a phenome-wide analysis (PheWAS) by using Gene ATLAS, which includes 778 diseases-traits from the UK Biobank [41]. Genes identified as candidates for drug inhibitors were included in this analysis (*CTSK, MAP3K11, KCNH2, ATP1B1, CAMK1D, PDGFD, IPO9, GGCX, CETP*). The strongest instrumental variable (lowest *P*-value) in MR or the variant prioritized in colocalization analyses for each of the potential drug target gene was assessed in the cross-phenotype association analysis. Diseases-traits were deemed significantly associated with the variant at FDR 5% and data are presented in Additional file 15: Table S15. In GTEx, gene variant A-rs12042263 (strongest IV in MR for the expression) (EUR freq = 0.91) is associated with a lower vascular expression of *CTSK* (*P*_eQTL_ = 1.69 × 10^–10^) and a decreased risk for CAD (OR: 0.96, 95% CI 0.95–0.98, P_GWAS_ = 1.18 × 10^–3^). In PheWAS, A-rs12042263 is positively associated with the risk of cerebrovascular disease (*P*_PheWAS_ = 3.15 × 10^–4^) (I67 other cerebrovascular disease) (Additional file 15: Table S15). These data suggest that the inhibition of CTSK may lower the risk of CAD (data in MR are concordant as the vascular expression of *CTSK* is positively associated with CAD-risk), but at the expense of increasing cerebrovascular events. Several drugs under investigation are reported in DGIdb to inhibit *CTSK* (Additional file 14: Table S14). A recent randomized clinical trial evaluating odanacatib, a CTSK inhibitor developed for postmenopausal osteoporosis, has found that inhibition of the target lowered primary endpoints, but increased the risk of stroke (HR 1.32, *P* = 0.03) [42]. Also, inhibition of *ATP1B1,* a gene positively associated with the CAD-risk in MR, is predicted to increase the risk of venous thromboembolic disease (Additional file 15: Table S15). Prediction based on colocalization analysis suggests that inhibition of *GGCX* may lower CAD-risk, but according to the PheWAS it is associated with an increase risk of malignant neoplasm of prostate (*P*_PheWAS_ = 3.06 × 10^–9^) (Additional file 15: Table S15). We next interrogated the Human Phenotype Ontology database [43] to identify disease-trait associations for the genes deemed druggable (Additional file 16: Table S16). In Human Phenotype Ontology, *CTSK*, *GGCX* and *KCNH2*, have been linked to bone-related conditions, coagulation defects and ventricular arrhythmia respectively. *KCNH2* encodes for the Ether-A-Go-Go-Related Protein 1, a potassium voltage-gated channel, involved in arrhythmia. Consistently, in the Side Effect Resource (SIDER) [44], several drugs targeting *KCNH2* in DGIdb are reported to induce ventricular arrhythmia. After a comprehensive filtering based on multiple resources including a PheWAS analysis and interrogation of Human Phenotype Ontology as well as SIDER databases, potential vascular drug targets such as *MAP3K11*, *CAMK1D*, *PDGFD*, *IPO9* and *CETP* were not predicted to be associated with major adverse side effects (cardiovascular, neurologic, metabolic, cancer) that would result from drug inhibition. Thus, some of these genes may represent suitable potential drug targets for follow-up studies.

### Impact of targeting MAP3K11 in vascular smooth muscle cells

**Fig. 5:**
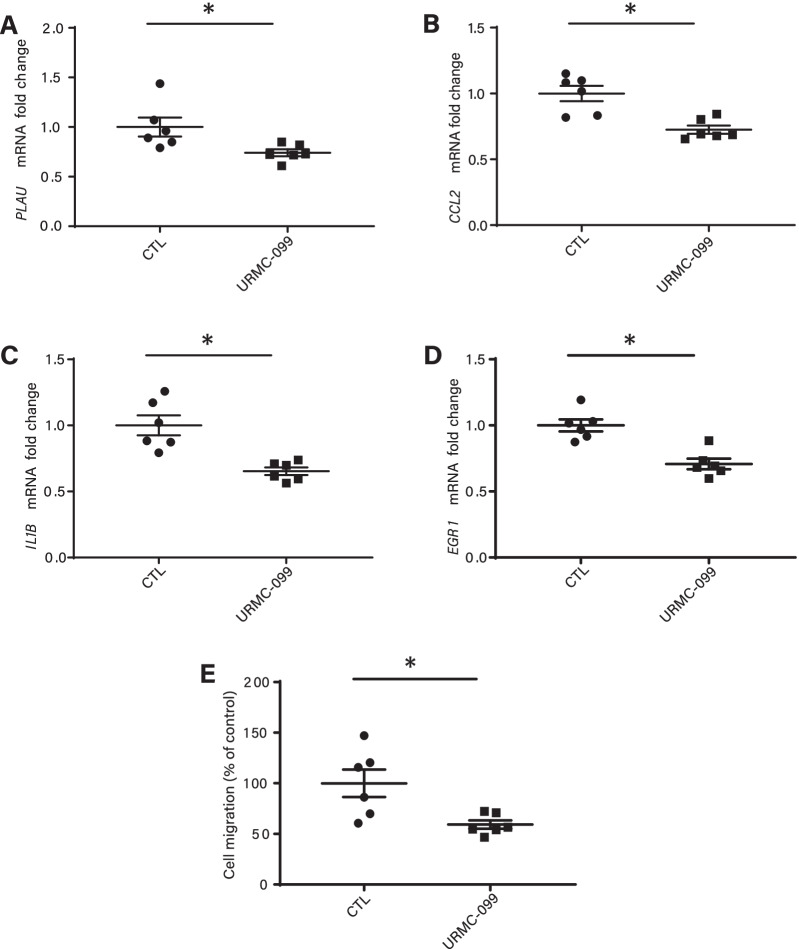
Functional impact of MAP3K11 inhibition on HCASMC. **A–D** VSMC treated with an inhibitor of MAP3K11 (URMC-099, 100 nM for 6 h) and expression mRNAs encoding for *PLAU*, *CCL2*, *IL1B* and *EGR;* *n* = 6. **E** Cell transmigration assay with URMC-099 (100 nM for 6 h); *n* = 6. The *n* represents experiments performed from 3 different donors in duplicate (performed at different passage); *P < 0.05

By using the present framework, we showed that some causally associated vascular genes were central in a network and were potentially druggable. Among those genes, *MAP3K11* (also known as *MLK3*) is positively associated with the CAD risk (OR: 1.07, 95%CI: 1.05–1.10, *P*_causal_ = 8.46 × 10^–9^) and is a target of the experimental compound URMC-099 [45]. Community network analysis using random walks showed that a module including *MAP3K11* (*P* = 1.94 × 10^–6^) (Additional file 20: Figure S2) was enriched in GO for the regulation of JNK cascade (*P* = 1.81 × 10^–10^) (Additional file 17: Table S17), which is involved in inflammation and cell migration [46]. URMC-099 is an experimental compound that inhibits MAP3K11. We hypothesized that URMC-099 may modulate the expression of key cytokines and transcription factors involved in the development of atherosclerosis. We observed that vascular smooth muscle cells (VSMCs) treated with URMC-099 (100 nM) for 6 h had lower expression of transcripts encoding for interleukin1 beta (*IL1B*), C–C motif chemokine ligand 2 (*CCL2* and also known as *MCP1*) and plasminogen activator urokinase (*PLAU*) (Fig. 5A–C). IL1B, CCL2 and PLAU are key mediators involved in plaque activity [47–49]. Early growth response 1 (EGR1) is a transcription factor known to enhance the expression of IL1B and chemokines as well as to promote the development of atherosclerosis in mice [50]. In isolated VSMCs, URMC-099 reduced the transcript level encoding for *EGR1* by 30% (Fig. 5D). Considering the pathway enrichment of CAD gene variants in cell migration (Additional file 2: Table S2), we assessed the impact of URMC-099 on VSMC transmigration in a Boyden chamber. Consistent with the impact of URMC-099 on genes having a role on cell migration such as *CCL2* and *EGR1* [51], we observed a reduction of cell migration by 40% in VSMC treated with the inhibitor for 6 h (Fig. 5E). As a proof of concept, these data provide further evidence that the pharmacological inhibition of predicted causally associated genes such as *MAP3K11* in VSMC impacts athero-relevant gene expression and cell phenotype.

## Discussion

In this work, we provide evidence that promoter-enhancer anchor loops in HCASMC explain 22% of the heritability for CAD. 3D mapped vascular smooth muscle genes were enriched in eQTL genes and in predicted causal targets. After filtering by using cross-phenotype analyses and curation of Human Phenotype Ontology and SIDER databases, we identified a set of druggable vascular genes, which are not predicted to be associated with major adverse events. Network of CAD-causally associated vascular genes was enriched in TGF-beta signaling, HDAC class I and MAPK pathways. As an illustrative case, the pharmacological inhibition of causally associated gene *MAP3K11* in vascular smooth muscle cells reduced cell migration, a key process involved in the development of plaque [52].

Among the causal candidate vascular genes identified by MR, *ARRB1* (OR: 0.84, 95% CI 0.78–0.91, *P*_causal_ = 9.45 × 10^–6^) and *MFGE8* (OR: 1.13, 95%CI: 1.09–1.17, *P*_causal_ = 2.08 × 10^–12^) were the genes with the highest effect size on the risk of CAD. *ARRB1* encodes for arrestin beta 1, which is involved in the regulation of G-protein coupled receptor (GPCR) signaling. As illustrated by the network approach, ARRB1 interacts with a large number of proteins and modulates, in a context specific manner, a myriad of signaling events related, among others, to inflammation [53]. At the 15q26.1 locus, we found a shared signal between the vascular expression of *MFGE8* and the risk of CAD (PP = 0.90). Taken together, colocalization and MR analyses strongly militate for a role of *MFGE8*, which encodes for Milk Fat Globule EGF And Factor V/VIII Domain Containing (also known as lactadherin), on the risk of CAD. A previous study conducted in VSMCs showed that the knockdown of *MFGE8* reduced the proliferation rate of cells [54]. Similarly, in a mouse model of vascular injury, the silencing of *MFGE8* decreased the migration of VSMCs and the formation of neointima [55]. Taken together, these data are consistent with an implication of *MFGE8* on the proliferation/migration of VSMC, a key process in atherogenesis.

Among the causal associations with CAD, 15 genes were identified by enhancer-promoter looping including 6 genes that were mapped only by using 3D genome data. Among those genes, several novel causal candidates, which have not been investigated in the context of atherosclerosis, were identified and include *EXOSC5, B3GNT8, ARRB1, PIF1, UTP11, AGPAT4* and *MAP3K11*. For instance, MR data indicate that the vascular expression of *AGPAT4* is negatively associated with CAD-risk. *AGPAT4* encodes for a membrane enzyme that inactivates lysophosphatidic acid, a small lipid mediator with pro-atherogenic activity [56]. Also, *PIF1*, which encodes for a helicase involved in the activity of telomerase [57], is a candidate gene for further exploration as the telomere length has been linked to the CAD-risk [58].

Network provides a holistic approach in identifying pathways and gene modules with specialized functions in chronic disorders [59]. A pathway analysis of the network constructed by using the candidate causal genes showed an enrichment for TGF-beta and MAPK pathways. These data are consistent with a role of the TGF-beta pathway in atherogenesis [60]. Drug targets are enriched in hub molecules [61] and the present data are in line with this notion. In this regard, we found that causally associated vascular genes were acting as hub in a network and were also deemed druggable according to DGIdb. After a comprehensive assessment of potential side effects, we narrowed down the number of druggable causal candidate genes to 5 targets (*MAP3K11*, *CAMK1D*, *PDGFD*, *IPO9* and *CETP)*. *PDGFD* was identified in colocalization analysis. The prioritized SNP, a frequent gene variant (T-rs974819, EUR freq = 0.72) was associated with an increased risk of CAD and a higher expression of *PDGFD* in the aorta. *PDGFD* encodes for a platelet derived growth factor with implication in atherosclerosis [62]. *CETP*, which has been identified by colocalization analysis, encodes for cholesteryl ester transfer protein. *CETP* is involved in the metabolism of high-density lipoprotein (HDL) and low-density lipoprotein (LDL). Genetic signal at the *CETP* locus suggested that inhibition of this enzyme may lower the risk of CAD [63]. Drugs targeting CETP were evaluated in 4 different randomized clinical trials with inconsistent results, which are possibly linked to drug-related off-target effects on the blood pressure and the design of studies (reviewed in [64]). In the present work, we identified by using MR that two druggable kinases (*MAP3K11*, *CAMK1D*) were positively associated with CAD. A previous analysis using MR showed that the expression of *CAMK1D* in blood cells was positively associated with the risk of CAD (OR: 1.05) [16]. These data are concordant with the present findings, which demonstrate a causal association for the vascular expression of *CAMK1D* on the risk of CAD (OR: 1.11). As a proof of concept, we evaluated a drug under development, which targets MAP3K11, on the expression of key genes involved in atherosclerosis and on the migration of VSMCs. In VSMCs, drug-induced inhibition of MAP3K11 reduced the expression of *IL1B* and *CCL2*, two genes causally associated with CAD [65, 66]. Also, the pharmacological inhibition of MAP3K11 decreased substantially the migration of VSMC, a cardinal process in the development of atheromatous plaques.

The present work has some limitations as causal inference using MR is subject to horizontal pleiotropy (i.e. gene variants that affect the outcome through an alternative pathway) [67]. However, the assessment of pleiotropy with the Cochran’s Q test and also the implementation of sensitivity analyses with the median weighted analyses minimize this risk. Also, the assessment of both MR and colocalization provides robustness to the findings. As such, the combined evidence derived from colocalization and MR for a drug target (gene) increased substantially the likelihood for a drug to be licensed [68]. Functional assays were carried out from a limited number of cell donors and additional follow-up studies are needed to probe the role of candidate causal vascular genes in atherosclerosis.

## Conclusions

In this work we found that the connectome of enhancer-promoter in HCASMC explained a significant proportion of the heritability for CAD. The mapping of genes using 3D enhancer-promoter contacts was enriched in vascular eQTL genes and causally-associated CAD genes. Among the causal candidate vascular genes, some are druggable and need further investigation to assess their role as potential pharmacologic targets for CAD.

## Methods

### CAD genetic associations

Summary statistics from GWAS data of a meta-analysis including 122,733 CAD cases and 424,528 controls from the UK Biobank and CARDIoGRAMplusC4D were downloaded for analyses [28]. GWAS meta-analysis was adjusted for age, gender and the first 30 principal components and included 7,947,838 gene variants. Cases from UK Biobank were identified by using ICD codes I21-I25 for ischemic heart disease and the corresponding OPCS-4 codes K40-K46, K49, K50 and K75 including percutaneous angioplasty. Patients reporting cardiovascular events such as myocardial infarction, coronary angioplasty and coronary artery bypass grafting were also included.

### Pathway analysis of genetic association data

Genetic association data for CAD were analyzed by using DEPICT, which provides an integrative approach to assess the most likely causal genes at risk loci and to infer pathway and tissue-cell enrichments [69]. Plink was used to identify independent loci based on GWAS significant SNPs (*P*_GWAS_ < 5E-8) and DEPICT was used with the prioritization of genes, gene sets and tissue-cells.

### Mapping the GWAS

Genetic association data for CAD was mapped by using the Functional Mapping and Annotation of GWAS (FUMA) framework [70]. Genomic risk loci were defined using a pre-calculated LD structure of the 1000G EUR reference population. SNPs in genomic loci with LD *r*^2^ < 0.6, *P*-value < 5 × 10^–8^ and MAF ≥ 0.01 were identified as independent significant SNPs (IndSigSNPs). IndSigSNPs independent from each other (LD *r*^2^ < 0.1) were identified as lead SNPs. Genomic loci closely located (< 250 kb based on the most right and left SNPs of each locus) were merged into one genomic locus. Gene annotation was based on Ensembl (build 85) and entrez ID yielding identification of 19,436 protein coding genes. Vascular eQTL (GTEx v8, aorta) were mapped by using IndSigSNPs to genes in *cis* (± 500 kb). SNP eQTL gene pairs were deemed significant at FDR 5%.

### Analysis of HiChIP

H3K27ac-HiChIP FASTQ files (GSE101498) from HCASMC were downloaded and aligned with HiC-Pro using the default settings [71]. Loop call was performed with FitHiChIP [72] at FDR < 1 × 10^–6^ and a resolution of 5 kb. Mapping of SNP to gene promoters was performed by using bedtools with the intersect function. Gene promoters were identified as a region ± 2 kb from the transcription start site (TSS) by using GENCODE version 35 in build 37. 1D H3K27ac-HiChIP track was generated using deepTools bamCoverage from the sorted BAM file generated by HiC-Pro. Hub enhancers were identified from H3K27ac-HiChIP by generating an interaction matrix of interacting pairs, which was analyzed with Cytoscape (version 3.8.2). Most connected hub enhancers were defined as those with a degree ≥ 90th percentile.

### Analysis of ATAC-seq and ChIP-seq

ATAC-seq and H3K27ac ChIP-seq data from HCASMC were downloaded (GSE124011). FASTQ files were extracted from SRA by using parallel-fastq-dump. Data were aligned on hg19 using Bowtie 2 and converted to bam files with Samtools. Duplicate reads were removed with Picard tools. Peak call was performed with MACS2 for broad peak with cutoff at 0.01. BigWig files were generated with deepTools bamCoverage and the sorted bam files.

### Enrichment analysis of anchor loops with open chromatin

Anchor loops and ATAC-seq bed files were analyzed for enrichment of overlap by using the R package GenometriCorr [73]. The projection test, which uses a binomial distribution was used to assess significance of calculated enrichment. Relative distance between the reference and query features were quantified by the density function correlation and illustrated in a heatmap.

### Pathway analysis of anchor loops

Significant loop calls of interacting pair regions in HCASMC H3K27ac-HiChIP identified from FitHiChIP were transformed into a bed file, which integrated the interacting regions or anchor loops. Enrichment was performed by using HOMER and the KEGG pathway.

### Partitioned heritability

Partitioned heritability of anchor loops in HCASMC was integrated to the full baseline model of 53 annotations in the stratified LD score regression framework [30]. LD score was calculated for each chromosome. CAD summary statistics was converted using the munge_sumstats.py and partitioned heritability calculated using the –h2 flag.

### Genetic Colocalization

Shared genetic signal between the eQTL and CAD was assessed by using the HyPrColoc package, which provides Bayesian colocalization analysis between traits [74]. Genomic regions were defined as 500 kb down- and 500 kb up-stream of the transcription start site (TSS) of each gene. Shared genetic signal between the expression (eQTL) and CAD was considered if the posterior probability PP > 0.8. Data were visualized by using LocusCompare.

### Mendelian randomization

Causal inference was evaluated with two-sample MR by selecting independent SNPs (instrumental variables) associated with the expression. SNPs were analyzed within a window of 500 kb around the TSS of each gene, then only SNPs strongly associated with the expression (*P* < 0.001 corresponds to ~ *F* statistics > 10) and independent (*r*^2^ < 0.1 based on the 1000G EUR reference panel) were selected as instrumental variables. MR was performed by using the Mendelian Randomisation package and a minimum of three instrumental variables was required to perform the analysis. Horizontal pleiotropy was estimated by using the Cochran’s Q test and was considered significant when *P*_heterogeneity_ < 0.05. Genes were considered causally associated with CAD at FDR 5% using the IVW MR. Sensitivity analysis was performed with the weighted median MR.

### Network analysis

Candidate causal genes were used to extract a network from the DifferentialNet dataset [39]. Data inferred for the aorta were extracted and analyzed by using NetworkAnalyst [75]. Centrality indices were downloaded from NetworkAnalyst and hub nodes (genes) were identified as those with a degree ≥ 90th percentile. Community identification was performed by using the random walk algorithm as implemented in NetworkAnalyst. Enrichment analysis was performed in Enrichr with the BioPlanet 2019 resource.

### Druggable genome

The Drug Gene Interaction Database (DGIdb) was leveraged to assess the druggability of candidate causal genes [40]. Drug-gene pairs identified in DGIdb were evaluated as potential candidate by using the directional effect of the expression on the CAD risk and whether the compound was identified as an agonist or antagonist (in case of enzyme: an inhibitor).

### PheWAS analysis

PheWAS analysis was performed in Gene ATLAS, which integrates 778 traits computed form 452,264 individuals in the UK Biobank [41]. Traits and disorders were deemed significantly associated with a SNP at FDR 5%.

### Cell culture experiments

Human aorta smooth muscle cells were obtained from patients undergoing heart transplantation. All donated tissues have been obtained with an explicit written consent approved by the local ethical committee and the investigations conducted in accordance with the Helsinki Declaration. Aortic roots were cut, the tunica adventitia and tunica intima were removed, and tissues were cut into pieces and incubated 8 min in Trypsin (Invitrogen, Thermo Fisher Scientific, ON, Canada) at 37 °C under agitation. Trypsin was then removed and tissues were resuspended in complete DMEM media (DMEM, 20% FBS with L-glutamine and sodium pyruvate). After cell growth (approximatively 1 month), DMEM media was removed and cells were cultured in smooth muscle cell basal media with growth supplements (#310-470 and #311-GS, Cell Application, CA, USA). Cells were used between passages 2–5. Cells were treated with 100 nM of URMC-099 (MedKoo Biosciences, NC, USA) as indicated in the result section. The expression of *IL1B*, *CCL2*, *PLAU* and *EGR1* was evaluated by quantitative real-time PCR. RNA from cells was isolated with E.Z.N.A. Micro RNA kit (Omega Bio-tek, VWR, QC, Canada). One μg of RNA was reverse transcribed using the Qscript cDNA supermix from Quanta (VWR, QC, Canada). qPCRs were performed with perfecta sybr supermix from Quanta on the Rotor-Gene 6000 system (Corbett Robotics Inc, CA, USA). Primers for *CCL2* were obtained from Qiagen (ON, Canada) and *IL1B*, *PLAU* and *EGR1* from IDT (IDT, IL, USA) (Additional file 18: Table S18). Transmigration assay was carried out in Boyden chamber with the QCM Chemotaxis Cell Migration Assay, 24-well (5 µm), fluorimetric (Millipore, Burlington, USA). 10,000 cells in DMEM 0% FBS with 100 nM of URMC-099 or with the control were loaded into the insert, and DMEM 10% FBS was applied outside the insert. Cell migration was quantified after 6 h according to manufacturer’s instructions.

### Statistics

For cell analyses, continuous data were expressed as mean ± SEM. Normality was tested with the Shapiro–Wilk test. Data with normal distribution were compared with Student *t*-test. For data with non-normal distribution, groups were compared with the Wilcoxon–Mann–Whitney test. Statistical analyses were performed with Prism 8.0.2. Hypergeometric and binomial tests were performed by using R version 4.0. FDR was calculated by using the R package multtest with the Benjamini and Hochberg test.

### URLs

Summary statistics GWAS CAD: https://data.mendeley.com/datasets/gbbsrpx6bs/1

GEO DataSets: https://www.ncbi.nlm.nih.gov/gds

GTEx v8: https://www.gtexportal.org/home/

DEPICT: https://data.broadinstitute.org/mpg/depict/

PLINK: http://zzz.bwh.harvard.edu/plink/

FUMA: https://fuma.ctglab.nl/

HiC-Pro: https://github.com/nservant/HiC-Pro

FitHiChIP: https://ay-lab.github.io/FitHiChIP/

parallel-fastq-dump: https://github.com/rvalieris/parallel-fastq-dump

Bowtie 2: http://bowtie-bio.sourceforge.net/bowtie2/index.shtml

Samtools: http://www.htslib.org/

Picard: https://broadinstitute.github.io/picard/

MACS2: https://pypi.org/project/MACS2/

deepTools: https://deeptools.readthedocs.io/en/develop/index.html

GenometriCorr: http://genometricorr.sourceforge.net/

HOMER: http://homer.ucsd.edu/homer/

Partitioned heritability: https://github.com/bulik/ldsc

HyPrColoc: https://github.com/jrs95/hyprcoloc

LocusCompare: http://locuscompare.com/

MR: https://cran.r-project.org/web/packages/MendelianRandomization/index.html

NetworkAnalyst: https://www.networkanalyst.ca/

Cytoscape: https://cytoscape.org/

DGIdb: https://www.dgidb.org/

Gene Atlas: http://geneatlas.roslin.ed.ac.uk/

## Supplementary Information

**Additional file 1.** Supplemental Table 1: DEPICT genetic association for CAD.

**Additional file 2.** Supplemental Table 2: DEPICT genetic association in pathways.

**Additional file 3.** Supplemental Table 3: Pathway enrichment for anchor loops in HCASMCs (KEGG).

**Additional file 4.** Supplemental Table 4: heritability of cis-anchor loops.

**Additional file 5.** Supplemental Table 5: SNP-enhancer-promoter loop pairs.

**Additional file 6.** Supplemental Table 6: SNP-eQTL gene pairs.

**Additional file 7.** Supplemental Table 7: Genetic colocalization with CAD.

**Additional file 8.** Supplemental Table 8: gene ontology enrichment for genes with colocalization.

**Additional file 9.** Supplemental Table 9: Mendelian randomization of 3D and eQTL mapped genes.

**Additional file 10.** Supplemental Table 10: gene ontology enrichment of genes causally associated with CAD.

**Additional file 11.** Supplemental Table 11: potential causal vascular genes for CAD.

**Additional file 12.** Supplemental Table 12: sensitivity analysis of causal vascular genes for CAD.

**Additional file 13.** Supplemental Table 13: network gene ontology enrichment.

**Additional file 14.** Supplemental Table 14: druggable genes based on DGIdb.

**Additional file 15.** Supplemental Table 15: diseases-traits significantly asociated with variants.

**Additional file 16.** Supplemental Table 16: diseases-traits associated with druggable genes.

**Additional file 17.** Supplemental Table 17: gene ontology for the module including MAP3K11.

**Additional file 18.** Supplemental Table 18: primer sequences.

**Additional file 19.** Supplemental Figure 1: Individual significant SNPs and 3D mapping in enhancer-promoter HiChIP.

**Additional file 20.** Supplemental Figure 2: Community network analysis.

## Data Availability

All the data are available in the manuscript including in the supplementary tables and figures. GWAs summary statistics for CAD and vascular eQTL (GTEx v8, aorta) as well as FASTQ files from GEO dataset were publicly available. Accession number and URLs are provided in the manuscript methods section.

## Abbreviations

CAD: Coronary artery disease
eQTL: Expression quantitative trait loci
GWAS: Genetic association studies
HCASMC: Human coronary artery smooth muscle cells
IVs: Instrumental variables
LD: Linkage disequilibrium
MR: Mendelian randomization
PheWAS: Phenome-wide analysis study
RCTs: Randomized clinical trials
DEPICT: Data-driven expression prioritized integration for complex traits
KEGG: Kyoto encyclopedia of genes and genomes
SNPs: Single nucleotide polymorphisms
GO: Gene ontology
IVW: Inverse variance weighted
FDR: False discovery rate
DGIdb: The drug gene interaction database
SIDER: Side effect resource
VSMCs: Vascular smooth muscle cells
HDL: High-density lipoprotein
LDL: Low-density lipoprotein
