# Supplementary material for: Enhancer promoter interactome and Mendelian randomization identify network of druggable vascular genes in coronary artery disease
Source: Hum Genomics. 2022 Mar 4;16:8. doi: 10.1186/s40246-022-00381-4 (PMC8895522; doi:10.1186/s40246-022-00381-4)
Supplement: Supplementary file 19 — Additional file 19. Supplemental Figure 1: Individual significant SNPs and 3D mapping in enhancer-promoter HiChIP. [file 40246_2022_381_MOESM19_ESM.pdf]

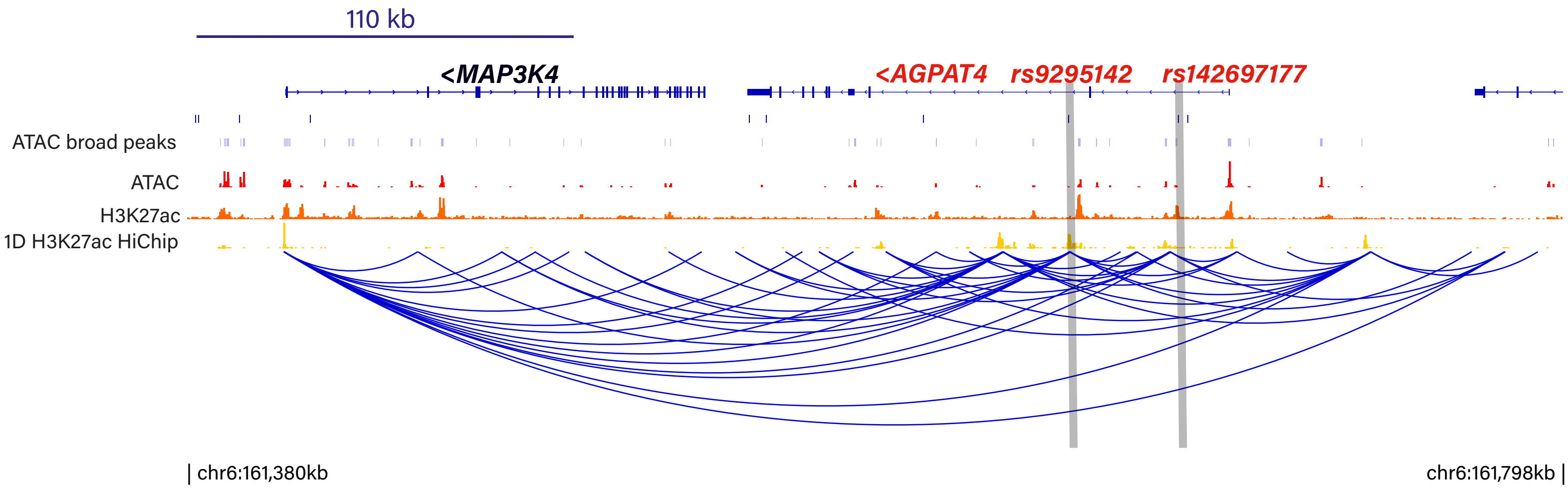

Suppl. Figure 1: Individual independent significant SNPs and 3D mapping in enhancer-promoter HiChIP; tracks represent genes, ATAC-seq, H3K27ac ChIP, 1D H3K27ac-HiChIP and arcs of significant loops; vertical bars represent the SNPs of interest and mapped genes are in red.
