# Supplementary figures and images for: Enhancer promoter interactome and Mendelian randomization identify network of druggable vascular genes in coronary artery disease
Source: Hum Genomics. 2022 Mar 4;16:8. doi: 10.1186/s40246-022-00381-4 (PMC8895522; doi:10.1186/s40246-022-00381-4)

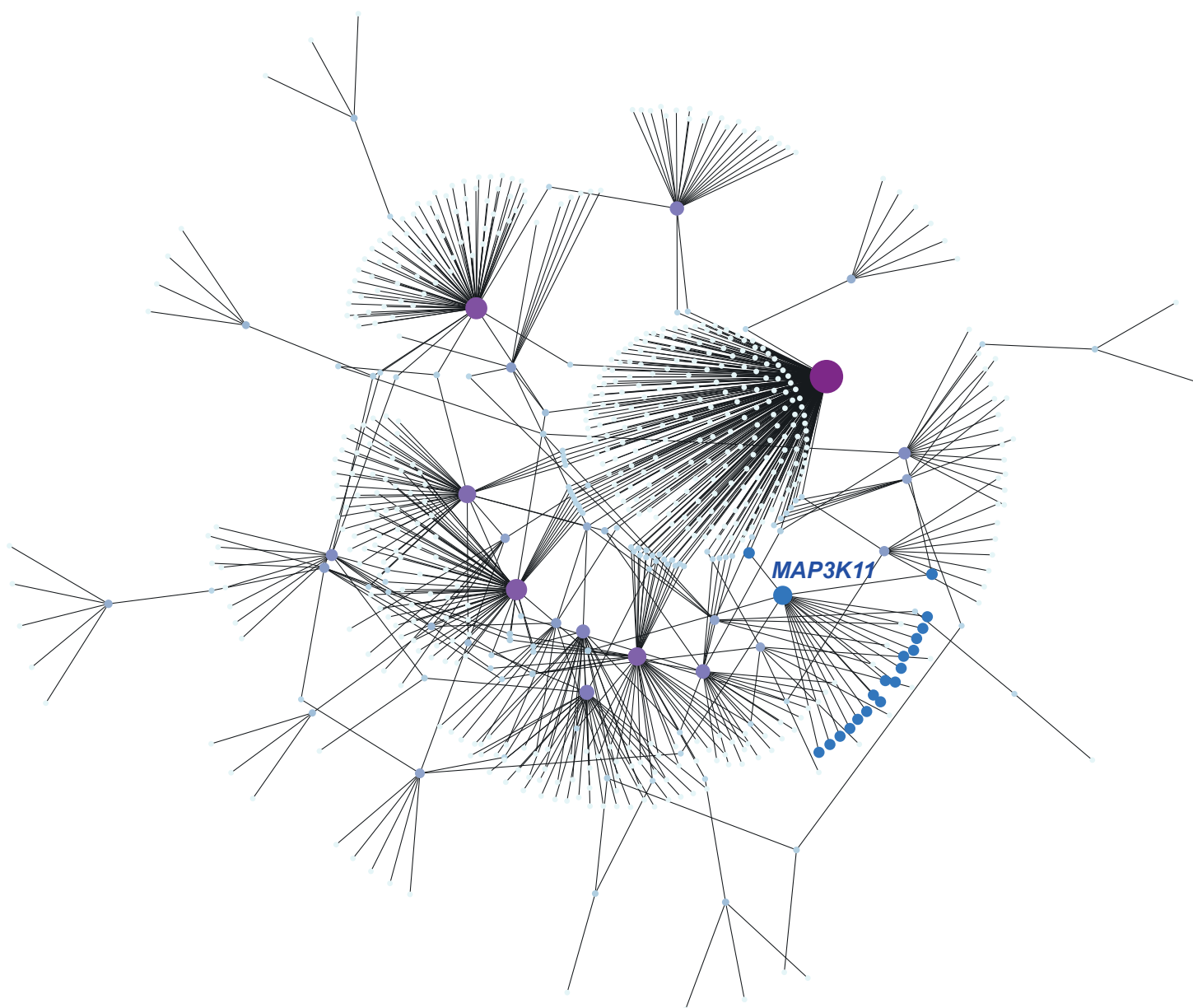

**Suppl. Figure 2:** Community network analysis. In blue, network community including MAP3K11 ( $P=1.94 \times 10^{-6}$ )

Supplement: Supplementary file 20 — Additional file 20. Supplemental Figure 2: Community network analysis. [file 40246_2022_381_MOESM20_ESM.pdf]
